# Age-based targeting of biannual azithromycin distribution for child survival in Niger: an adaptive cluster-randomized trial protocol (AVENIR)

**DOI:** 10.1186/s12889-021-10824-7

**Published:** 2021-04-29

**Authors:** Kieran S. O’Brien, Ahmed M. Arzika, Abdou Amza, Ramatou Maliki, Sani Ousmane, Boubacar Kadri, Beido Nassirou, Alio Karamba Mankara, Abdoul Naser Harouna, Emily Colby, Elodie Lebas, Zijun Liu, Victoria Le, William Nguyen, Jeremy D. Keenan, Catherine E. Oldenburg, Travis C. Porco, Thuy Doan, Benjamin F. Arnold, Thomas M. Lietman, the AVENIR Study Group, Nicolas De Borman, Stephane Dresse, Romain Olekhnovitch, Grégoire Lurton, Léa Monchy, Martin De Wulf, Rebecca Brander, Dennis Chao, James Heine, Rasa Izadnegahdar, Laura Lamberti, Assaf Oron, Surabhi Rajaram, Matthew Steele, Amina Seyfoule, Ahmed Arzika, Fati Bello, Diallo Boubacar, Amadou Harouna, Alio Karamba, Ramatou Maliki, Farissat Nomao, Abraham Omar, Ibrahima Issa, Ronan Jambou, Rabiou Labbo, S’ Hooshim N. Lamine, Sani Ousmane, Boubakar Rakia, Maikano Sadikou, Brianna Lindsay, Nupur Kittur, David Roos, Sheena Tomko, Issa Adji, Djibo Ali, Souleymane Alzouma, Maidanda Boubacar, Diegou Boureima, Cheik Boureima Daouda, Idi Moussa Djatao, Ibrahim Jean Etienne, El Hadji Boubakar H. Maiga, Amadou Oumarou, Ocquet Sakina, Sanoussi Samuila, David Addiss, Mourtala Assao, Julia Bielicki, Allen Hightower, Brian Jackson, Fiona Russell, Frances Hocking, Zied Mhirsi, Dan Pawson, Agbessi Amouzou, Robert Black, Waqas Ahmed, Todd D. Hatajik, Julie Jenson, Chuck Knirsch, Chiao-Chin Lin, Amza Abdou, Nassirou Beido, Boubacar Kadri, Boubacar Maïdanda, Maelle Ba, Yaye Sophiétou Diop, Yacine Djibo, Gráinne Hutton, Fara Ndiaye, Benjamin Arnold, Cindi Chen, Emily Colby, Catherine A. Cook, Thuy Doan, Jeremy D. Keenan, Victoria Le, Elodie Lebas, Thomas M. Lietman, Zijun Liu, William Nguyen, Kieran S. O’Brien, Catherine E. Oldenburg, Travis C. Porco, Kevin Ruder, George W. Rutherford, Lina Zhong, Zhaoxia Zhou, Stefano M. Bertozzi, Jim G. Khan, Elliot Marseille, Gianluca Boo, Edith Darin, Andy Tatem

**Affiliations:** 1 Francis I. Proctor Foundation, University of California, San Francisco, USA; 2 Centre de Recherche et Interventions en Santé Publique, Birni N’Gaoure, Niger; 3 Programme Nationale de Santé Oculaire, Niamey, Niger; 4 Centre de Recherche Médical et Sanitaire, Niamey, Niger; 5 Department of Ophthalmology, University of California, 490 Illinois Street, San Francisco, CA 94158 USA; 6 Department of Epidemiology and Biostatistics, University of California, San Francisco, USA; 7 Institute for Global Health Sciences, University of California, San Francisco, USA

**Keywords:** Azithromycin, Mortality, Cluster-randomized trial, Adaptive trial, Mass drug administration

## Abstract

**Background:**

Biannual distribution of azithromycin to children 1–59 months old reduced mortality by 14% in a cluster-randomized trial. The World Health Organization has proposed targeting this intervention to the subgroup of children 1–11 months old to reduce selection for antimicrobial resistance. Here, we describe a trial designed to determine the impact of age-based targeting of biannual azithromycin on mortality and antimicrobial resistance.

**Methods:**

AVENIR is a cluster-randomized, placebo-controlled, double-masked, response-adaptive large simple trial in Niger. During the 2.5-year study period, 3350 communities are targeted for enrollment. In the first year, communities in the Dosso region will be randomized 1:1:1 to 1) azithromycin 1–11: biannual azithromycin to children 1–11 months old with placebo to children 12–59 months old, 2) azithromycin 1–59: biannual azithromycin to children 1–59 months old, or 3) placebo: biannual placebo to children 1–59 months old. Regions enrolled after the first year will be randomized with an updated allocation based on the probability of mortality in children 1–59 months in each arm during the preceding study period. A biannual door-to-door census will be conducted to enumerate the population, distribute azithromycin and placebo, and monitor vital status. Primary mortality outcomes are defined as all-cause mortality rate (deaths per 1000 person-years) after 2.5 years from the first enrollment in 1) children 1–59 months old comparing the azithromycin 1–59 and placebo arms, 2) children 1–11 months old comparing the azithromycin 1–11 and placebo arm, and 3) children 12–59 months in the azithromycin 1–11 and azithromycin 1–59 arms. In the Dosso region, 50 communities from each arm will be followed to monitor antimicrobial resistance. Primary resistance outcomes will be assessed after 2 years of distributions and include 1) prevalence of genetic determinants of macrolide resistance in nasopharyngeal samples from children 1–59 months old, and 2) load of genetic determinants of macrolide resistance in rectal samples from children 1–59 months old.

**Discussion:**

As high-mortality settings consider this intervention, the results of this trial will provide evidence to support programmatic and policy decision-making on age-based strategies for azithromycin distribution to promote child survival.

**Trial registration:**

This trial was registered on January 13, 2020 (clinicaltrials.gov: NCT04224987).

**Supplementary Information:**

The online version contains supplementary material available at 10.1186/s12889-021-10824-7.

## Background

Despite an overall decrease in the burden of childhood mortality in sub-Saharan Africa, as many as 10% of children do not survive to the age of 5 years in some areas [1, 2]. To achieve the Sustainable Development Goal (SDG) to end preventable under-5 mortality by 2030, parts of western and central Africa must achieve unprecedented rates of mortality reduction [2, 3]. With estimates at 108 deaths per 1000 livebirths [1], under-5 mortality in Niger is more than 4 times the SDG target of 25 deaths per 1000 livebirths [3]. In such high mortality settings, effective and feasible interventions are urgently needed.

Azithromycin distribution has been proposed as a stop-gap intervention to reduce the burden of preventable deaths in high mortality settings while health systems are strengthened. Trachoma programs have provided oral azithromycin to entire communities for decades to prevent blindness [4], with more than 900 million doses distributed globally [5]. The potential for azithromycin distribution to reduce child mortality was discovered in randomized controlled trials conducted for trachoma [6–8]. The Macrolides Oraux pour Réduire les Décès avec un Œil sur la Résistance trial (MORDOR) later demonstrated that biannual distribution of oral azithromycin to children 1–59 months of age reduced mortality in this age group by 13.5% in Malawi, Niger, and Tanzania (95% CI 6.7 to 19.8) [9]. The greatest mortality reductions were observed in Niger (18.1% reduction, 95% CI 10.0 to 25.5) and among the youngest children (24.9% reduction in children 1–5 months old, 95% CI 10.6 to 37.0), subgroups which also had the highest baseline mortality rates [9]. Although the exact mechanism of effect remains unclear, evidence suggests that azithromycin distributions reduce the burden of respiratory infections, diarrhea, and malaria [10], common causes of under-5 mortality in sub-Saharan Africa [1].

Despite the potential for this intervention to improve child survival, concern about selection for antimicrobial resistance (AMR) underpins caution in implementation. Evidence from trachoma studies and the MORDOR trial indicate that azithromycin distribution increases the community prevalence of macrolide resistance in respiratory and enteric organisms [11, 12]. An increase in AMR could decrease the efficacy of macrolides or other antibiotics against target pathogens, although longer-term evidence from MORDOR did not find evidence of waning efficacy against all-cause mortality, even with an increase in genetic determinants of AMR [13]. Trachoma programs, which distribute azithromycin to everyone in a community as opposed to just children, suggest that resistance decreases once distributions are discontinued [12].

The World Health Organization (WHO) released conditional guidelines that specify targeting azithromycin distributions to children 1–11 months of age in high mortality settings [14]. The rationale for targeting 1–11 months is that the youngest children experience the highest risk of mortality and limiting distributions to a subgroup of children could reduce selection for antimicrobial resistance. However, distributing azithromycin to children 1–11 months alone remains untested, since MORDOR I treated children 1–59 months of age. A crude examination of relative mortality reductions by age group suggested a stronger effect in the younger age groups, but MORDOR was unable to demonstrate a differential effect by age group statistically [9]. Moreover, a herd effect could have contributed to the apparent larger effect sizes in the younger groups; treating children 12–59 months may protect children 1–11 months by reducing their exposure to infectious diseases causing mortality. Finally, in MORDOR-Niger there were 5 times as many children aged 12–59 months as children 1–11 months, resulting in nearly twice as many deaths averted in the older age group [15]. The greatest societal gains may thus come from intervening on a population with a broader risk spectrum instead of targeting high-risk subgroups [16, 17]. More evidence is needed to determine the magnitude of and difference in survival benefit when targeting different age groups, and of the magnitude of AMR selection when distributing to different age groups.

The AVENIR trial (Azithromycine pour la Vie des Enfants au Niger: Implementation et Recherche) was designed to test the effect of age-based targeting on mortality and resistance. Several design features support these objectives. First, as mortality is a rare event even in high mortality settings, a large sample size is required to detect modest intervention effects [18]. AVENIR thus uses a large simple trial design, with a simple intervention, minimal data collection, and simple outcome assessment allowing for feasible implementation in a very large population. Second, AVENIR is cluster randomized both because of the community-based nature of the intervention and to address potential contamination within communities [19]. As the mechanism of azithromycin’s effect is likely through its impact on infectious disease, community-level randomization prevents an individual’s treatment assignment from affecting the outcomes of individuals with different assignments within a community. Finally, AVENIR will use response-adaptive allocation to achieve several aims. This approach allows for more ethical allocation, as communities will have a higher probability of receiving the intervention that reduces mortality the most. By maintaining an allocation of at least 10% of communities to placebo, continued monitoring of efficacy is still possible. This includes the detection of waning effects over time which might indicate the appropriate duration of the intervention. The adaptive design also allows for greater flexibility, enabling the existing trial infrastructure to be used as a platform to evaluate other interventions [20]. The study can adapt to changing guidelines by randomizing in new intervention arms, for example, rather than being constrained by the original treatments which could become irrelevant during the course of the trial due to a change in policy recommendations.

## Methods/design

### Study design overview

**Fig. 1:**
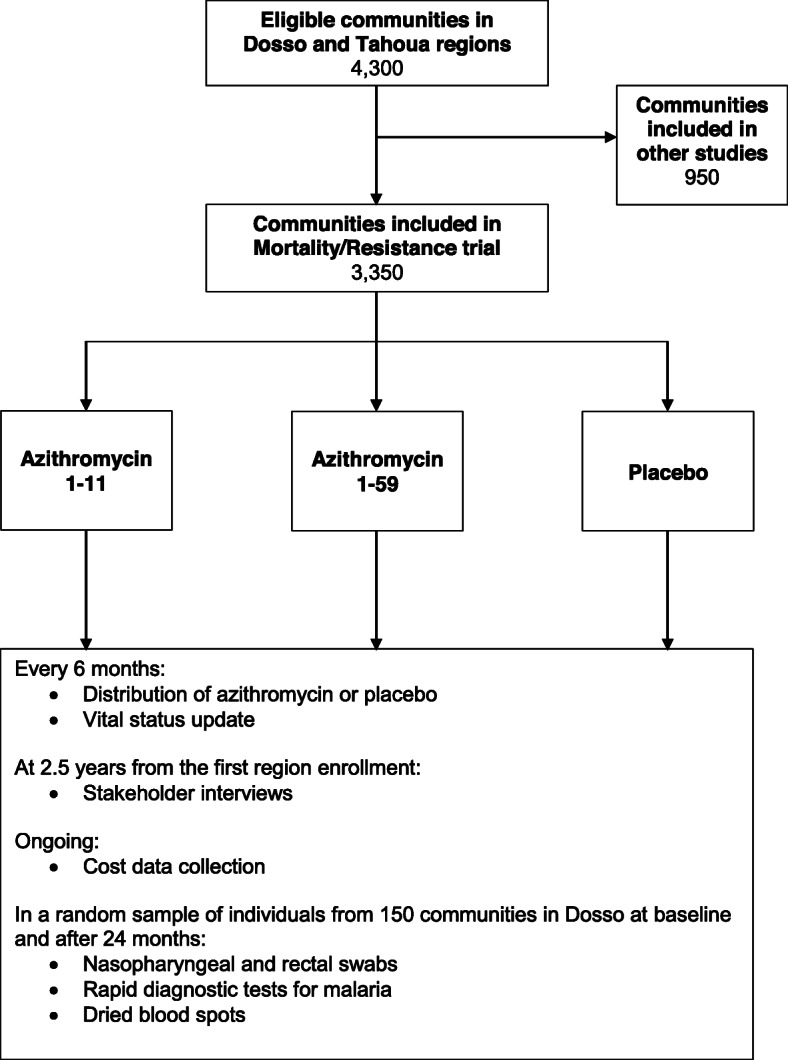
AVENIR trial design summary for communities included in primary outcomes during Stage I. Communities randomized to the azithromycin 1–11 arm will receive biannual distribution of azithromycin to children 1–11 months of age with placebo to children 12–59 months of age. Communities randomized to the azithromycin 1–59 arm will receive biannual distribution of azithromycin to children 1–59 months of age. Children randomized to the placebo arm will receive biannual distribution of placebo to children 1–59 months of age

AVENIR is a double-masked cluster-randomized placebo-controlled response-adaptive large simple trial in Niger. During a one-year run-in period, communities in the Dosso region in Niger will be randomized 1:1:1 to 1) **azithromycin 1–11:** biannual azithromycin to children 1–11 months old with placebo to children 12–59 months old, 2) **azithromycin 1–59:** biannual azithromycin to children 1–59 months old, or 3) **placebo:** biannual placebo to children 1–59 months old. Regions enrolled after the run-in period will be randomized with an allocation updated based on the probability of mortality in children 1–59 months in each arm during the preceding study period. Communities will retain their allocation for 4 distributions, after which they will be re-randomized with an updated allocation. All-cause mortality will be compared by arm at 2.5 years from the first enrollment. In a substudy, 50 randomly selected communities from each arm in the Dosso region will be followed to monitor antimicrobial resistance. Figure 1 summarizes the mortality and resistance trial designs for the Dosso and Tahoua regions.

The trial began enrollment in November 2020 and recruitment is anticipated through May 2023. Stage I includes enrollment through the primary mortality analysis and Stage II includes potential continued enrollment after this time point. This protocol adheres to the Standard Protocol Items: Recommendations for Interventional Trials (SPIRIT) guidelines (Supplementary Table S1, [21]) and refers to version 13 of the study protocol as revised 31 January 2021.

### Objectives and hypotheses

The objectives of this study are 1) to compare the efficacy of two age-based approaches to biannual mass azithromycin distribution to reduce all-cause mortality, 2) to compare selection for antimicrobial resistance across these strategies, and 3) to evaluate implementation-related outcomes, including the costs, feasibility, and acceptability of this intervention, and to compare these indicators across distribution strategies. Specifically, objective 1 has 3 aims: 1) to replicate and assess the generalizability of the MORDOR trial by comparing 1–59-month mortality in the azithromycin 1–59 and placebo arms, 2) to test the efficacy of targeting treatment to children 1–11 months old by comparing 1–11-month mortality in the azithromycin 1–11 and placebo arms, and 3) to determine the additional benefit of including older children by comparing 12–59-month mortality in the azithromycin 1–11 and azithromycin 1–59 arms. We hypothesize that mortality will be lower in both azithromycin arms compared to placebo, and lower in the azithromycin 1–59 arm compared to the azithromycin 1–11 arm at 2.5 years from the first enrollment. For objective 2, the aims are 1) to compare the community-level prevalence of genetic determinants of macrolide resistance in respiratory organisms across arms and 2) to compare the community-level load of genetic determinants of macrolide resistance in enteric organisms across arms. We hypothesize that, after 2 years of distributions, resistance will be higher in both azithromycin arms compared to placebo, and higher in the azithromycin 1–59 arm compared to azithromycin 1–11. Objective 3 involves the calculation of program costs for each strategy, analysis of the comparative cost-effectiveness, and the measurement of feasibility and acceptability as perceived by stakeholders at the community, implementer, and governmental levels. We hypothesize that the azithromycin 1–59 arm will be more cost-effective than the azithromycin 1–11 arm and that these strategies will be similarly feasible and acceptable.

### Setting, participants, and eligibility

**Fig. 2:**
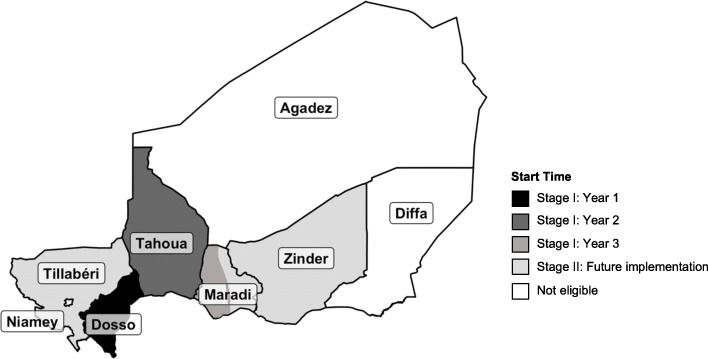
Eligible regions in Niger with and AVENIR study timeline. Stage I includes implementation in the Dosso, Tahoua, and first half of the Maradi regions, with communities from Dosso and Tahoua contributing to the primary outcomes described in this protocol. Continuation to Stage II depends on the primary outcome results as well as logistical, financial, and security concerns. Stage II would include implementation in the remaining half of Maradi as well as the Zinder and Tillabéri regions. The map was created by the authors in R (R Foundation for Statistical Computing, Vienna, Austria)

Full eligibility criteria at the community and individual levels are described in Table 1. This study will include secure and accessible rural and peri-urban communities in the 5 regions of Niger with the largest under-5 populations (Dosso, Tahoua, Maradi, Zinder, Tillabéri; Fig. 2, Table 2), with Stage I targeting the Dosso, Tahoua, and the first half of the Maradi regions for inclusion. Dosso and Tahoua regions will contribute to the primary outcomes in Stage I. The estimated 2017 regional under-5 mortality rates were 116 per 1000 live births in Dosso, 101 in Tahoua, 110 in Maradi, 120 in Zinder, and 116 in Tillabéri [22]. We estimate that these 5 regions include approximately 14,630 eligible communities encompassing approximately 2.3 million eligible children under 5 (Table 2) and 3350 of these are targeted for inclusion in Stage I.

**Table 1:** Eligibility criteria at the community- and individual-levels for the AVENIR mortality and resistance Stage I trial in Niger

Trial	Level	Eligibility Criteria
Mortality	Community	• Location in Dosso or Tahoua region • Population between 250 and 2499^a^ • Distance > 5 km from district headquarters town • Not designated as a “quartier” on national census • Accessible and safe for study team • Verbal consent from community leader(s)
	Individual	• Primary residence in a study community • Age 1–59 months • Weight ≥ 3.0 • No known allergy to macrolides • Verbal consent of guardian for study participation
Resistance	Community	• Location in Dosso • Accessible and safe for study team • Randomly selected • Not included in MORDOR trials • Verbal consent of community leader(s)
	Individual	• Primary residence in a selected study community • 1–59 months old, 7–12 years old or guardian of child eligible for treatment • Randomly selected from census • Verbal consent from guardian

^a^ Population size as estimated from the most recent national census or projections

**Table 2:** Estimated eligible population and study timeline by region in Stages I and II of the AVENIR trial

Region	Estimated eligible under-5 population^c^	Estimated eligible communities^d^	Stage I^a^					Stage II^b^		
			Year 1		Year 2		Year 3	Year 3	Year 4	
			Round 1	Round 2	Round 3^e^	Round 4	Round 5	Round 6	Round 7	Round 8
Dosso	271,273	1800	X*	X	X	X	X*	X	X	X
Tahoua (1st half)	245,944	1250			X	X	X	X	X	X
Tahoua (2nd half)	245,944	1250				X	X	X	X	X
Maradi (1st half)	342,889	1700					X	X	X	X
Maradi (2nd half)	342,890	1700						X	X	X
Zinder	452,241	3860							X	X
Tillabéri	440,791	3070								X
**Total**	**2,341,972**	**14,630**								

Round = one distribution of azithromycin and/or placebo; x = census and treatment; x* = census, resistance assessments, treatment

^a^ Stage I includes enrollment through the primary outcome analysis at 2.5 years from the first region enrollment

^b^ Stage II is dependent upon primary outcome results, available resources, and security restrictions

^c^ As estimated from projections by the Institute for Health Metrics and Evaluation multiplied by fraction of eligible grappes in each region

^d^ Communities with total populations between 250 and 2499 not designated as urban quartiers according to the 2012 Niger national census. Dosso region estimates further exclude communities known to be located within 5 km of district headquarters town

Distance-based exclusions pending for the other 4 regions.

^e^ Interim analysis to be conducted at 18 months from the first enrollment

### Recruitment

AVENIR was designed in collaboration with local, national, and global stakeholders in child health. Sensitization campaigns will be conducted at the community, district, regional, and national levels before study activities begin and leaders at these levels will continue to be engaged by the study team throughout the course of the trial and in the dissemination of results. All households will be recruited for participation in the census, and all children 1–59 months of age will be recruited for the intervention.

### Randomization and masking

The randomization unit will be the *grappe,* which is an administrative unit defined during the 2012 general population census in Niger, hereafter referred to as “community.” The randomization sequence will be generated by one trial biostatistician and data analyst in R (R Foundation for Statistical Computing, Vienna, Austria).

Implementation will be phased in by region (Fig. 2, Table 2). Communities in Dosso will be enrolled in the first year and randomized to the 3 arms in a 1:1:1 allocation. After 1 year, allocation probabilities for newly enrolled communities will adapt using the probability that each arm is superior with respect to the mortality rate in children 1–59 months, such that communities will have a higher probability of being allocated to the arm with the lowest mortality rate. From the pool of eligible communities in the Dosso region, a substudy will include a random sample of 50 communities per arm to monitor AMR outcomes.

After the primary outcome analysis at 2.5 years from the first enrollment, communities may be re-randomized with continued follow-up using the same adaptive allocation. To minimize the impact of a possible carry-over effect of treatment in the event that a community changes arms through adaptive allocation, each community will stay with its randomized assignment for 4 distributions (approximately 2 years). No community will be re-randomized by the time of the primary outcome analysis. Additional treatment arms may be added to the trial or arms may be dropped from the trial after the primary outcome analysis. With such changes, protocol details, power calculations, and analysis plans will be updated accordingly.

Masking will be facilitated by use of a matching placebo, which will be identical to azithromycin in appearance, smell, packaging, and distribution. Those masked to study arm allocation include participants, investigators, and most study personnel including census personnel administering treatment and collecting mortality outcomes and laboratory personnel processing samples for resistance outcomes. Unmasked personnel include the trial biostatistician and data analyst responsible for implementing the randomization sequence and key members of Pfizer staff responsible for implementation of the randomization sequence.

### Interventions

Azithromycin and placebo will be administered biannually as a single, directly observed dose of 20 mg/kg (up to the maximum adult dose of 1 g) as oral suspension using dosing cups or syringes. For children 1–11 months of age and those unable to stand, dose will be determined by weight using a hanging scale. For children 12–59 months of age able to stand, dose will be determined by height approximation as currently performed by Niger’s trachoma program [23]. Azithromycin and placebo will be prepared by Pfizer, Inc. (New York, NY, USA) and shipped directly to the study sites.

### Outcome assessments

#### Mortality

A door-to-door census will be conducted every 6 months to enumerate households, identify eligible children, administer azithromycin and placebo, and monitor vital status. At the household level, GPS coordinates will be taken and demographic data will be recorded for the head of household, caregivers, and children 1–59 months old. For eligible children, weight and/or dose will be recorded along with treatment adherence. At subsequent census periods, study teams will return to each household to update the vital status of children, indicating whether a child is alive, has died, has moved, or has an unknown status. New children and households will be added. The census will be updated at approximately 6, 12, 18, 24, 30, and 36 months from baseline.

The primary mortality outcome is the all-cause mortality rate (deaths per 1000 person-years at risk) at 2.5 years from the first enrollment, with a window of up to 3 months. Inter-census intervals will be used to determine mortality rates. Children will be included as died in the primary outcome if vital status is recorded as alive at one census and died at the subsequent census. Person-time at risk will be calculated as time alive and eligible for treatment while living in the study area, with children who died, moved, or have an unknown status contributing half of the person-time for their last inter-census interval. Three primary comparisons will be made: 1) mortality rate in children 1–59 months in the azithromycin 1–59 vs placebo arms, 2) mortality rate in children 1–11 months in the azithromycin 1–11 vs placebo arms, and 3) mortality rate in children 12–59 months in the azithromycin 1–11 vs azithromycin 1–59 arms.

Secondary mortality outcomes include mortality rate in children 12–59 months compared between the azithromycin 1–11 and placebo arms and mortality rate in children 1–11 months compared between the azithromycin 1–11 and azithromycin 1–59 arm. Subgroups of interest include region, number of past distributions, distribution timing and seasonality, compliance, anthropometric indicators, and presence of other interventions like seasonal malaria chemoprevention or trachoma distribution of azithromycin. Weight as measured to determine dosage will also be compared by arm over time.

#### Antimicrobial resistance

A random sample of 50 communities per arm will be selected from eligible communities in the Dosso region and followed to monitor antimicrobial resistance and other outcomes. In each of the selected communities, 30 children aged 1–59 months, 30 children aged 7–12 years, and 30 caregivers/guardians of eligible children will be randomly selected from the census for additional monitoring. Nasopharyngeal samples will be collected from all selected individuals. From children 1–59 months, rectal swabs, rapid diagnostic tests for malaria, and dried blood spots will also be collected. Children with positive RDT results will be referred for further care. Environmental samples will also be taken from selected communities and households, including community-level water sources and latrines, and household-level stored water, animal feces, and dirt. Samples will be collected at baseline before the first distribution and after 4 distributions (approximately 2 years). Genotypic methods will be used to process samples to determine the presence of genetic determinants of resistance to macrolides and other classes of antibiotics, including targeted PCR for the nasopharyngeal swabs and metagenomic deep sequencing for the rectal swabs at the community level. In the sample of children 1–59 months old, 10 children will be randomly selected for an additional nasopharyngeal swab which will be used for phenotypic testing of resistance in *Streptococcus pneumoniae*.

The primary AMR outcomes include 1) the prevalence of genetic determinants of macrolide resistance in children 1–59 months old from nasopharyngeal swabs*,* and 2) the load of genetic determinants of macrolide resistance in children 1–59 months old from rectal swabs.

Secondary outcomes from these collections include resistance to other classes of antibiotics among samples taken from children 1–59 months old, the proportion of macrolide-resistant pneumococcal isolates from nasopharyngeal swabs in children 1–59 months old, indirect effects of azithromycin distribution on resistance to macrolides and other classes of antibiotics among untreated individuals living in the same household as those eligible for treatment (children 7–12 years and adults), genetic determinants of antibiotic resistance in environmental samples, the prevalence of malaria in children 1–59 months old, and serological markers of pathogen infection.

#### Implementation outcomes

Program costs will be measured by macro-costing activities using routine administrative data collection and by focused micro-costing activities using both routine administrative data collection and detailed time and motion data collection. Cost-effectiveness analysis will assess the added net costs, added health outcomes, and the incremental cost-effectiveness ratio comparing cost per health outcome achieved between distribution strategies. These analyses will be assessed from different perspectives: households, community, health system, and society. Feasibility will be tracked using administrative and monitoring data collected on timeliness and fidelity of intervention rollout, training and supervision metrics, supply inventory and storage, and AMR monitoring. Acceptability of the program and the two distribution strategies will be assessed using semi-structured interviews on the perception of and satisfaction with the intervention, facilitators and barriers to participation and implementation, and relevance of the intervention to the interviewees. Interviews will be conducted with stakeholders at multiple levels, including participant, community leaders, implementers, and those involved in health system policy and programmatic decision-making.

### Adverse events

At the time of treatment, caregivers will be instructed to report any adverse event experienced by the treated child within 28 days of treatment to the study team. Serious adverse events will be reported to UCSF and the Medical Monitors within 24 h of notification, and to Pfizer, the Data and Safety Monitoring Committee (DSMC), and Institutional Review Boards (IRBs) according to the requirements of each. Children will be referred for follow-up care on a case-by-case basis.

### Data collection, management, and monitoring

All data will be collected electronically on mobile devices with a custom-designed mobile application (CommCare by Dimagi, Cambridge, MA, USA), and uploaded regularly to a secure, cloud-based server. All study personnel will attend intensive, two-day training sessions prior to conducting study procedures and collecting data. Data management teams in Niger and at UCSF will monitor data collection in real time daily. Investigators will visit study sites twice each year to monitor study implementation and ensure adherence to the study protocol.

Study participants will be assigned unique identification numbers that do not contain personal identifying information, which will be used to link data across databases and will be the only identifier shared in de-identified datasets. Upon completion of the trial, de-identified data will be made available upon request.

### Study oversight

A Data and Safety Monitoring Committee (DSMC) will be empaneled before the study begins to provide independent oversight of data quality and participant safety. The DSMC will include independent experts with collective expertise in bioethics, biostatistics, antimicrobial resistance, and pediatric infectious disease. The DSMC will meet at least once per year to review study progress and adverse events, with ad hoc meetings convened as necessary. The DSMC will recommend modifications to the protocol as necessary. Major protocol changes will be reported to the DSMC and Institutional Review Board(s) as required.

Study investigators from UCSF and in Niger will monitor study conduct regularly through data monitoring as described above in addition to regular site visits to ensure adherence to the study protocol. Details of ethical approval and informed consent are included below. Two Medical Monitors with expertise in pediatrics and infectious disease will review the protocol before the trial begins and will review serious adverse events during the trial.

### Statistical considerations

#### Adaptation

The adaptive allocation will use all outcome measurements collected by each time of adaptation. The algorithm involves fitting a negative binomial regression model with the count of deaths as the outcome, community-level person-time at risk during each inter-census interval as an offset, and indicators for each arm. The model will be used to estimate each arm’s log mortality rate and standard error. Then, 10,000 replicates of the log mortality rates will be drawn from the estimated distributions and the arm with the lowest mortality rate in each replicate will be determined. The probability that each arm has the lowest mortality rate will be estimated as the proportion of the 10,000 replicates in which the arm has the lowest mortality rate. To mitigate drastic swings in allocation due to change, a fourth-root transformation will be applied to the probabilities which was determined through simulation. To preserve the ability to make comparisons, no arm’s allocation probability will be allowed to fall below 10% [24].

#### Sample size and power

Power calculations were based on the primary mortality and resistance outcomes and the anticipated number of communities enrolled by 2.5 years (Table 2). In Stage I, 1400 communities will be randomly selected from the 1800 eligible communities in Dosso to participate for 2 full years (4 inter-census intervals), 975 communities in Tahoua will be selected from approximately 2500 eligible communities to participate for 1 full year (2 inter-census intervals), and another 975 communities in Tahoua will be selected from the eligible pool to participate for one half year (1 inter-census interval). In total, the primary outcome is expected to include 8525 inter-census intervals from 3350 communities (1116 per arm). Table 3 summarizes the detectable effect sizes and the assumptions used in the calculations.

**Table 3:** Detectable effect sizes and assumptions used in sample size calculations for mortality and resistance primary outcomes for the AVENIR trial

Trial	Outcome	Comparison	Power	Alpha	Baseline^a**,**b^	SD or ICC^b^	Number of communities per arm	Relative effect size	Absolute effect size
Mortality^c^	Mortality in 1–59-month group	Azithromycin 1–59 vs placebo	80%	0.05	27 deaths per 1000 person-years	0.019	1116	10%	2.7 per 1000 person-years
	Mortality in 1–11-month group	Azithromycin 1–11 vs placebo	80%	0.05 (if 1–59 group *P* < 0.05)	45 deaths per 1000 person-years	0.068	1116	19%	8.8 deaths per 1000 person-years
	Mortality in the 12–59-month group	Azithromycin 1–11 vs azithromycin 1–59	80%	0.05 (if 1–11 group *P* < 0.05)	24 deaths per 1000 person-years	0.018	1116	11%	2.7 deaths per 1000 person-years
Resistance^d^	Prevalence of genetic determinants, NP swabs in 1–59-month group	Azithromycin 1–11 vs placebo Azithromycin 1–59 vs placebo Azithromycin 1–11 vs 1–59	80%	0.05/2	30%	0.045	20	43%	13%
	Load of genetic determinants, rectal swabs in 1–59-month group^e^	Azithromycin 1–11 vs placebo Azithromycin 1–59 vs placebo Azithromycin 1–11 vs 1–59	80%	0.05/2	2.13	0.02	20	70%	0.74

*ICC* intra-class correlation coefficient, *SD* standard deviation

^a^ Refers to baseline mortality rate for mortality outcomes, and baseline prevalence or load of genetic determinants of macrolide

resistance for resistance outcomes.

^b^ Estimated using data from the MORDOR Niger trial, SD reported for mortality rates and ICC for resistance

^c^ Calculations based on standard Z-test formula for power

^d^ Calculations based on Eqs. 7.11 and 7.12 for prevalence and load, respectively [19].

^e^ Load of genetic determinants of resistance presented on the log2 scale

Sensitivity analyses were conducted to estimate power based on reduced enrollment caused by security or logistical concerns. If only the first half of Tahoua communities are enrolled by 2.5 years, resulting in 30% fewer communities overall (791 communities per arm), we would still have 80% power to detect the following relative effect sizes: 12, 23, and 13% for 1–59 months, 1–11 months, and 12–59 months, respectively.

#### Analysis of primary outcomes

The primary analysis for the mortality outcomes will be negative binomial regression with count of deaths in each community as the outcome and community-level person-time at risk during each inter-census interval as an offset. The models will include indicator variables for treatment arm and inter-census interval. The adaptive allocation will be accounted for using pooled regression, with each inter-census interval a stratum with its own allocation probabilities [24]. For each of the three primary mortality outcomes, the analysis will be subset to include the target age group. The parameter of interest estimated from each model is the incidence rate ratio (IRR), with 95% confidence intervals (Cis) estimated using bootstrap resampling at the community level. A fixed-sequence, hierarchical testing approach will be used for the three primary mortality outcomes [25], such that the 1–59-month mortality will be compared first. If that comparison between the azithromycin 1–59 and placebo arms is statistically significant at an alpha of 0.05, then testing proceeds to the second outcome, 1–11-month mortality. Similarly, if the comparison between azithromycin 1–11 and placebo arms is statistically significant at an alpha of 0.05, then testing proceeds to the third outcome. Regardless of hypothesis testing results, we will report 95% confidence intervals around estimates of the incidence rate ratio for each comparison. Effect heterogeneity will be assessed for pre-specified subgroups on the additive and multiplicative scales [26, 27].

The primary analysis for the AMR outcomes will be ANOVA to compare the community-level proportion or load of genetic determinants of resistance to macrolides across the three arms. *P-*values for all AMR analyses will be computed using a permutation test, with a step down minP procedure to control for three pairwise comparisons in each primary macrolide resistance outcome at an alpha of 0.05 [28, 29], and using a Benjamini-Hochberg correction to control the false discovery rate at 5% across additional (non-macrolide) endpoints [30]. For all AMR endpoints, we will additionally report unadjusted *P*-values, which will be less conservative to detect undesirable outcomes resulting from mass distribution of azithromycin.

All primary analyses will be intention-to-treat and hypothesis tests will be two-sided superiority tests. Permutation *P*-values will be estimated for each comparison [31]. Ongoing analyses after the primary endpoint will be conducted using a Bayesian perspective.

#### Interim analysis

An interim analysis will be conducted at 1.5 years after the first enrollment based on the Haybittle-Peto boundary, which results in an interim alpha of 0.001 and retains an alpha of 0.05 for the primary analysis [32]. Testing will proceed according to the hierarchical approach proposed for the primary analysis and will be conducted by the unmasked members to the data team. The DSMC will be notified of the results. If *P* < 0.001 for any comparison, the DSMC may consult with one of the investigators to determine a course of action while keeping the rest of the team masked.

## Discussion

Targeted approaches to azithromycin distribution have been proposed to focus benefits on subgroups at the highest risk of mortality while limiting the amount of antibiotics distributed, theoretically reducing the risks posed by the intervention. Based on the results of the MORDOR trial [9], the WHO released conditional guidelines supporting azithromycin distribution to children 1–11 months of age to promote child survival in high mortality settings in sub-Saharan Africa [14]. As targeting this age group alone has not been tested, the AVENIR trial aims to compare the effect of distributing azithromycin to children 1–11 months old versus children 1–59 months old on mortality, resistance, and implementation outcomes in a cluster-randomized placebo-controlled large simple trial with response-adaptive allocation in Niger.

AVENIR is designed to provide evidence to support decision-making on age-based strategies for distributing azithromycin to support child survival. The large simple trial design ensures adequate power to detect a modest intervention effect on mortality [18], which is a rare outcome even in this high mortality setting. To ensure feasibility of conducting a study on such a large scale, this design requires minimal data collection, which limits the ability to describe the study population in detail as well as to define subgroups. In addition, this design lacks the power to detect differential effects of the intervention on mortality outcomes among subgroups. The use of response-adaptive randomization allows for ethical allocation of interventions and the ability to use the trial platform to evaluate other interventions as well. AVENIR’s cluster-randomized design reduces the contamination likely with an individual randomized design in this setting while aligning with the community-based nature of azithromycin distribution [19]. The main sets of outcomes included in this trial allow for a comparison of the potential reduction in mortality across strategies, the risk of increasing antimicrobial resistance, and the feasibility and acceptability of implementation.

Equipoise is among the implications of the ethical complexity involved in balancing the potential benefit against the risks of this intervention [33]. Although MORDOR provided evidence of the efficacy of azithromycin for child survival in one setting, the risk of antimicrobial resistance warrants caution before widespread implementation is adopted. Additionally, MORDOR suggested that the mortality effect is dependent on baseline mortality rates [9, 34], so intervention efficacy could change over time with secular trends in mortality. Similarly, another trial was unable to demonstrate an effect of azithromycin distribution on mortality when administered alongside Seasonal Malaria Chemoprevention [35], indicating the effect may depend on the presence or absence of other child survival interventions. Further evidence of efficacy in other settings along with continued monitoring of resistance would support programmatic decision-making. Placebo-controlled trials are the most rigorous approach to assess the effects of this intervention on both mortality and antimicrobial resistance, as the potential for bias is great without a proper comparison group, particularly for mortality.

In conclusion, AVENIR will provide evidence on mortality, AMR, and implementation outcomes after different age-based azithromycin distribution strategies. These results will support policy and programmatic decision-making as high mortality settings consider implementation of this intervention to promote child survival. The trial infrastructure will further provide a platform from which to continue to evaluate community-based interventions on a large scale.

## Supplementary Information

**Additional file 1.**

## Data Availability

Trial results will be presented at local, national and international meetings and submitted to peer-reviewed journals for publication. De-identified trial data will be made publicly available after publication of primary outcomes.

## Abbreviations

AMR: Antimicrobial Resistance
AVENIR: Azithromycine pour la Vie des Enfants au Niger: Implementation et Recherche
DSMC: Data and Safety Monitoring Committee
MORDOR: Macrolides Oraux pour Réduire les Décès avec un Œil sur la Résistance
SDG: Sustainable Development Goal
UCSF: University of California, San Francisco
WHO: World Health Organization
